# 

**Published:** 1891-01

**Authors:** 


### CONTENTS.

COMMUNICATIONS.

Some Notes on Experimental Implantation of Teeth.................... 5
Chloride of Methyl.................................................... 20
Ohio State Dental Society............................................. 24
Dreams and Visions.................................................... 27
Hypnotism............................................................. 31
A Means of Holding the Rubber Dam while Operating upon Cavities in Labial and Buccal Surfaces.................................. 36

SELECTIONS.

Physiological Action and Uses of Cocaine.............................. 40
The Relation of Syphilis to the Practice of Dentistry................. 42
A Sound Body First.................................................... 44
Bacteriology Run to Extremes.......................................... 45
Improved Hypodermic Syringe........................................... 47
Oliver Wendell Holmes................................................. 49
To Save the Edge of Sterilized Instruments............................ 50

EDITORIAL.

Case of Impacted Third Molar.......................................... 50
Northern Ohio Dental Association...................................... 52
Aristol............................................................... 53
Bibliographical....................................................... 55
Mississippi Valley Dental Association................................. 55
Obituary—Dr. Bradley.................................................. 56

